# An experimental study of cathodic protection for chloride contaminated reinforced concrete

**DOI:** 10.1617/s11527-018-1273-1

**Published:** 2018-10-25

**Authors:** Hayder M. Oleiwi, Yu Wang, Michele Curioni, Xianyi Chen, Guowen Yao, Levingshan Augusthus-Nelson, A. H. Ragazzon-Smith, Igor Shabalin

**Affiliations:** 1 School of Computing, Science and Engineering, University of Salford, Manchester, M5 4WT UK; 2 School of Materials, Corrosion and Protection Centre, University of Manchester, Manchester, M13 9PL UK; 3 Charter Coating Service (2000) Ltd., Calgary, AB T2E 6P1 Canada; 4 School of Environment and Life Sciences, University of Salford, Manchester, M5 4WT UK; 5 School of Civil Engineering, Chongqing Jiaotong University, Chongqing, 400074 China; 6 College of Engineering, University of Thi-Qar, Nasiriyah, Iraq

**Keywords:** Cathodic protection, Concrete resistivity, Reinforcement corrosion, CP design criteria

## Abstract

Cathodic protection (CP) is being increasingly used on reinforced concrete structures to protect steel reinforcing bars from corrosion in aggressive conditions. Due to the complexity of environmental conditions, the design specifications in national and international standards are still open to discussion to achieve both sufficient and efficient protection for reinforced concrete structures in engineering practices. This paper reports an experimental research to investigate the influence of chloride content on concrete resistivity, rebar corrosion rate and the performance of CP operation using different current densities. It aims to understand the correlation between the chloride content and concrete resistivity together with the CP current requirement, and to investigate the precision of the CP design criteria in standards.

## Introduction

The corrosion of steel reinforcements has been recognised as the major cause for the premature deterioration of reinforced concrete structures worldwide [1]. Extensive researches on the deterioration mechanisms have concluded that the combined presence of chloride and the decrease in pH due to carbonation plays the most significant role in the corrosion of concrete reinforcements [2, 3]. So far, many technologies using chemical, mechanical, and electrochemical methods have been developed to address the problem [4, 5]. Among those, cathodic protection (CP), has been widely recognised and become the most popular technique implemented in civil engineering practices for its reliable long term protection [6–8].

Adequate protection provided by CP for the steel reinforcement in concrete depends on many factors. In addition to the steel composition and the nature of concrete components, the physical conditions, such as concrete porosity, degree of carbonation, water and chloride contents, and environmental temperature, play the important roles affecting the effectiveness of CP operation. CP arrangement and the applied current densities are all related to the above conditions [9, 10]. Additionally, the service life of the anode is another factor to be taken in consideration [11, 12]. Traditionally, titanium mesh sheet with noble metal oxides coating, such as iridium, ruthenium and cobalt, have been the most common type of anodes [12]. Other materials, offering ease of installation and cost efficiency, have also been employed [13]. In recent years, due to its good chemical stability, carbon fibre has been successfully used as anode material in CP implementation for concrete structures [13–15].

In general, there are two acceptable criteria in CP performance appraisal. One relates to the instant-off potential (the potential measured immediately when the CP system is switched off) of the reinforcement. The other one relates to the potential decay (depolarization) of the reinforcement [16, 17]. The specifications in national and international standards for the criteria were principally established on the empirical evaluation of the data obtained from successfully operated CP cases [18]. For example, Takewaka [19] suggested that the corrosion of reinforcement in concrete structures could be stopped when the potential of the rebars was less than − 600 mV with respect to Ag/AgCl/0.5KCl reference electrode. For chloride-contaminated concrete, more negative potentials in the range of − 645 to − 705 mV with respect to Ag/AgCl/0.5KCl were reported by Shi et al. [20]. British standard 12696:2012 [21] specifies that the instant-off potential should be more negative than − 720 mV with respect to Ag/AgCl/0.5KCl for any concrete structures. For the depolarization criterion, the widely adopted specification is that the reinforcement potential should decay (i.e. become less negative) by at least 100 mV over a period of 4–24 h starting from an ‘instant-off’ potential [18, 21, 22].

Applying an adequate current density to ensure sufficient current across the critical areas of the protected reinforcement [11] but at a cost efficient energy consumption and without overprotection is vital to avoide unnecessary expenses and the potential negative effect of the hydrogen production due to the activated cathodic reactions at the rebar and concrete interface. No CP implementation can achieve an effective protection using a specified constant current density throughout the life span of concrete structures [16]. A previous work suggested that, for newly built concrete structures, a current density in the range of 1–2 mA/m^2^ on the rebars is sufficient for protection, while for the structures that have already suffered from reinforcement corrosion, a current density in the range of 5–20 mA/m^2^ is recommended [23]. Higher practical CP current densities in the range of 30–50 mA/m^2^ were also suggested when reinforcements are exposed to severe environmental conditions [16].

Based on the discussion above, it is noted that some uncertainty still exist on the topic of defining the current specification for CP design for reinforced concrete structures for varied and complex application conditions. As an effort to obtain more detailed specific information for the CP design for chloride contaminated reinforced concrete structures, this paper reports an experimental study on the effect of concrete chloride contamination degree on the corrosion evaluation parameters that are employed for reinforcement cathodic protection assessment. Specifically, this work investigates the correlation between the chloride content and concrete resistivity, and the relationship of these two parameters with the rebar corrosion rate. These studies enable identification of more precise characteristic relationships between concrete chloride content, the applied current density and the instant-off potential. Thus, the experimental results provide a direct guidance for the specification of the CP current density requirements for atmospherically exposed concrete structure at different levels of chloride contamination.

## Specimens preparation

Concrete specimens used in this study were prepared following the method recommended by the British Building Research Establishment (BRE) [24] to give a 28 days compressive strength of 38 N/mm^2^. Locally produced limestone Portland cement (CEM II/A-LL in British standard BS EN 197-1: 2011) was used at 390 kg/m^3^. Natural sands of the maximum size of 4.75 mm and a specific gravity of 2.47 were used for the fine aggregates at 1125 kg/m^3^. The coarse aggregates were limestone of maximum size of 10 mm and a specific gravity of 2.49, and were used at 580 kg/m^3^ in proportion. Pure NaCl (0, 1, 2, 3.5, and 5% of the cement weight) was added as contaminant into the mix water, to prepare specimens with different chloride contamination contents. The concrete mixes had a water to cement ratio of 0.4.

**Fig. 1:**
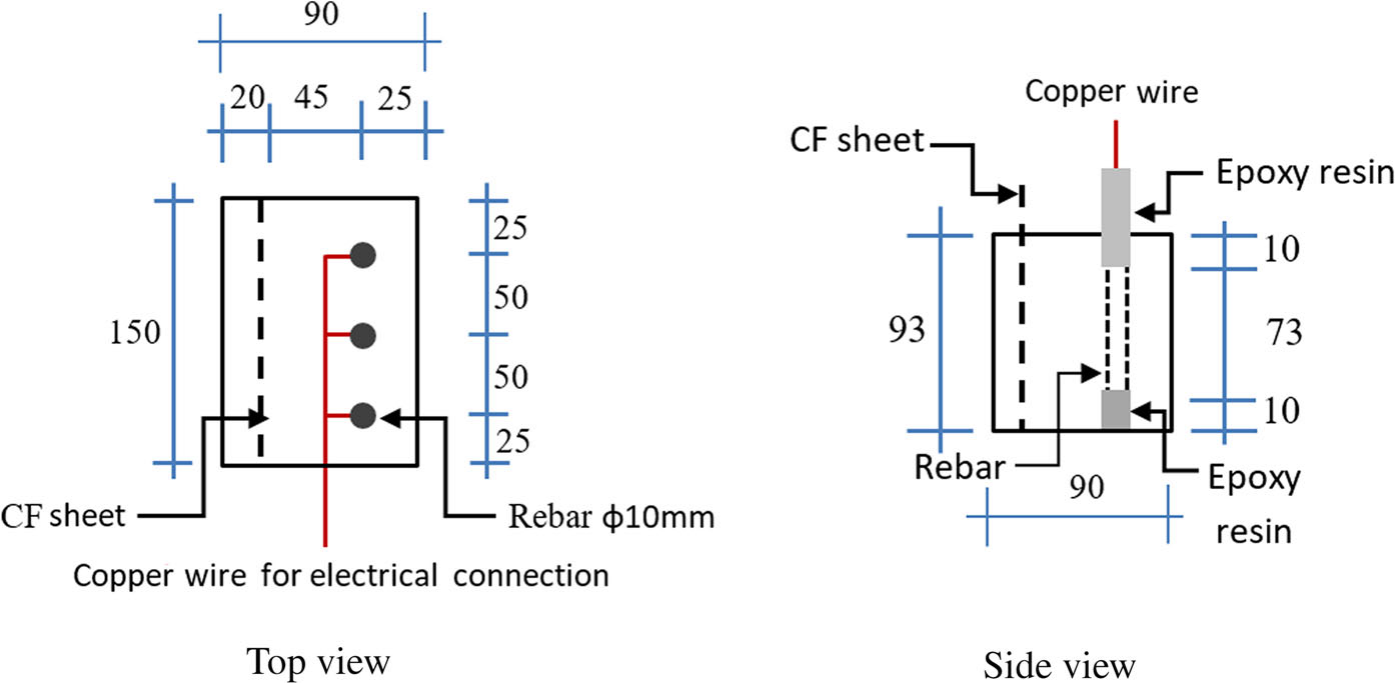
The configuration and dimension of the reinforced concrete specimens

Ten reinforced concrete specimens (2 specimens for each chloride content) with the size of Length × Height × Depth = 90 × 93 × 150 mm^3^, illustrated in Fig. 1, were used to investigate the CP operation. Each specimen had three conventional reinforcing bars of 10 mm diameter to simulate local rebar clusters in structures. On the other hand, considering that the rebars’ position in concrete affects the corrosion rate due to differences in oxygen access, taking an average response of the three electrically connected rebars aims to minimize the influence of location on the final results. A hole of 3 mm in diameter and 5 mm in depth was drilled at one end of each rebar. One end of a copper wire used for electrical connection and it was soldered into the hole for fully integrated contact. The two ends of all the steel rebars were coated using epoxy resin to prevent direct exposure to the environment when embedded in concrete specimens. The middle region of an effective length 73 mm along the rebar axis was directly exposed to the concrete environment to give a total exposed surface area of 3*π* × 10 × 73 = 6880 mm^2^. A layer of a woven (CF) sheet was embedded in each specimen to be used as the anode. The nominal surface area of the embedded carbon fibre anode is 144 × 93 mm^2^. The carbon fibre anode extended about 30 mm out of the specimens for electrical connection. All the cast reinforced concrete specimens had the entire part of the steel bars exposed to the atmosphere coated again using epoxy resin after the concrete set.

**Fig. 2:**
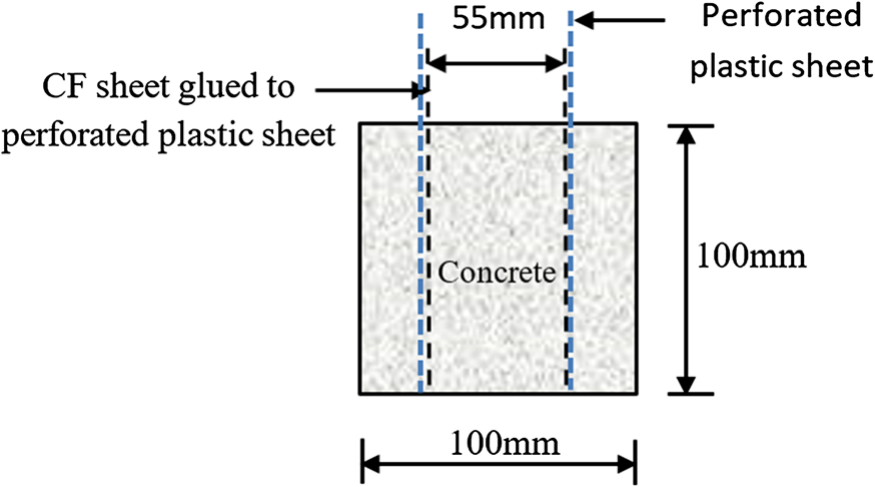
Illustration of concrete specimens used for electrical resistivity measurements

Another ten rectangular concrete specimens of the size of Length × Height × Depth = 100 × 100 × 70 mm^3^ and with two parallel embedded woven carbon fibre (CF) sheets as shown in Fig. 2, were prepared for concrete resistivity measurement using the same mixtures and the curing procedure as that of the reinforced concrete specimens described above. The two woven carbon fibre sheets are used for the electrodes, which were kept a fixed distance of 55 mm from each other and held in the upright position using two rigid perforated plastic plates in moulds when cast the concrete specimens [25].

All the prepared concrete specimens were placed in water with the same chloride concentration as that of the mix water used and cured for 28 days. Such method aims to ensure an even chloride distribution. Thereafter all concrete specimens were taken out and exposed to an atmosphere of a relative humidity of 50 ± 5% and a temperature of 20 ± 3 °C for 5 weeks, i.e. until they attained a stable weight before conducting all the experiments. To obtain the accurate total chloride contents in the specimens, another ten concrete specimens of all the same mixtures (two specimens for each designed chloride content) with the size of 100 × 100 × 100 mm^3^ and cured in the same conditions were analysed using potentiometric titration method described in ASTM C1152/C1152M-12 [26].

## Experimental methods

### Corrosion rate and concrete electrical resistivity

The corrosion rates of rebars in the reinforced concrete specimens were assessed before the implementation of CP using the linear polarization method described by Stern and Geary [27]. The potential of the three reinforcing bars were acquired together through the soldered wires. The corrosion rate of the three rebars was measured in terms of the average current density, *i*_corr_. A small potential shift, Δ*E*, was applied on rebars of an open circuit potential, *E*_corr_. The potential shift varied from − 20 to + 20 mV [28, 29] at a scan rate of 0.125 mV/s using a computer controlled Gamry potentiostat (Model 1000E). The IR drop was automatically compensated by the programmed potentiostat.

Polarization resistance, *R*_p_, was then determined according to the slope of the plot of the applied voltage versus the measured current at the point of zero current. The same method was applied for all the specimens for the sake of comparison [30]. The corrosion current was determined following the Stern-Geary equation,

1 $$ I_{\text{corr}} = \frac{B}{{R_{\text{p}} }} \times 10^{ - 3} , $$

where *B *= (*β*_*a*_*β*_*c*_/2.3(*β*_*a*_ +* β*_*c*_)) is a constant in mV, *β*_*a*_ and *β*_*c*_ are the anodic and cathodic Tafel constants, *R*_p_ is the polarization resistance in kΩ (Δ*E*/Δ*I*), *I*_corr_ is the corrosion current in mA.

A value of 26 mV was used for the constant *B* for the chloride contaminated specimens [6, 31] while 52 mV was used for the chloride free specimens. The corrosion rate, *i*_corr_, in (mA/m^2^) is determined in terms of the Eq. (2) [32], where *A* was the total exposure surface area of all the three rebars in a specimen.

2 $$ i_{\text{corr}} = \frac{{I_{\text{corr}} }}{A}. $$

Concrete electrical resistivity was measured using the two-electrode specimens (Fig. 2). A sinewave alternating current of 3000 mV amplitude and a frequency of 10 kHz was applied across the two parallel electrodes. The electrical resistivity of the concrete was calculated in terms of the revised form of Ohm’s law below [33].

3 $$ \rho = \frac{V}{I}\frac{A}{L}, $$

where ρ is electrical resistivity, *V* is the amplitude of the applied voltage, *I* is the amplitude of the measured current, A is the cross-section area of the concrete specimen perpendicular to the current flow or parallel to the two electrode plates, and L is the distance between the two electrodes.

### Cathodic protection

**Fig. 3:**
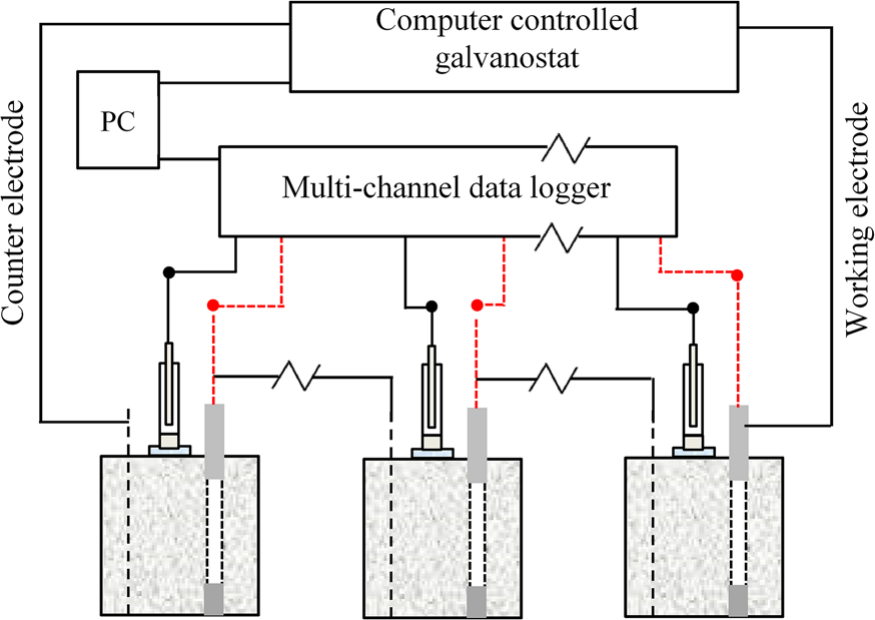
Experimental scheme for reinforced specimens under CP

Galvanostatic polarization technique was adapted to apply ten different CP current densities on the rebars in each specimen. They are 5, 10, 15, 20, 25, 35, 45, 55, 65, and 75 mA/m^2^, respectively. Ten specimens (two for each chloride content) were connected in series in each test at the same time as shown in Fig. 3. Silver/Silver chloride (Ag/AgCl/0.5KCl) half cells were used for the reference electrodes. Multi-channel data logger with 10,000 kΩ input impedance and 0.1 mV resolution was used for the collection of all potential readings.

Each test had a certain CP current density applied for 24 h and afterwards switched off for more than one day (24 h) to ensure a sufficient depolarization of the rebars. In the time, the potential of rebars was continuously recorded from the start and until 4 h after the interruption of the CP current in the time of depolarization. Based on the recorded data, the instant-off potential, and 4-h potential decay can be obtained.

## Results and discussion

### Chloride contents, corrosion rate and concrete resistivity

The measured chloride contents in the cured specimens of each mixes with different added NaCl and the corresponding electrical resistivity of the concrete are listed in the Table 1. Chloride contents are expressed in term of the percentage of the cement weight of specimens.

**Table 1:** Chloride content, reinforcement corrosion rate and concrete resistivity

Added NaCl % cement weight	0	1	2	3.5	5
Measured total Cl^−^ % cement weight	0	0.814	1.425	2.258	3.301
Concrete resistivity kΩ cm	18.4	15.9	12.4	10.1	7.7

**Fig. 4:**
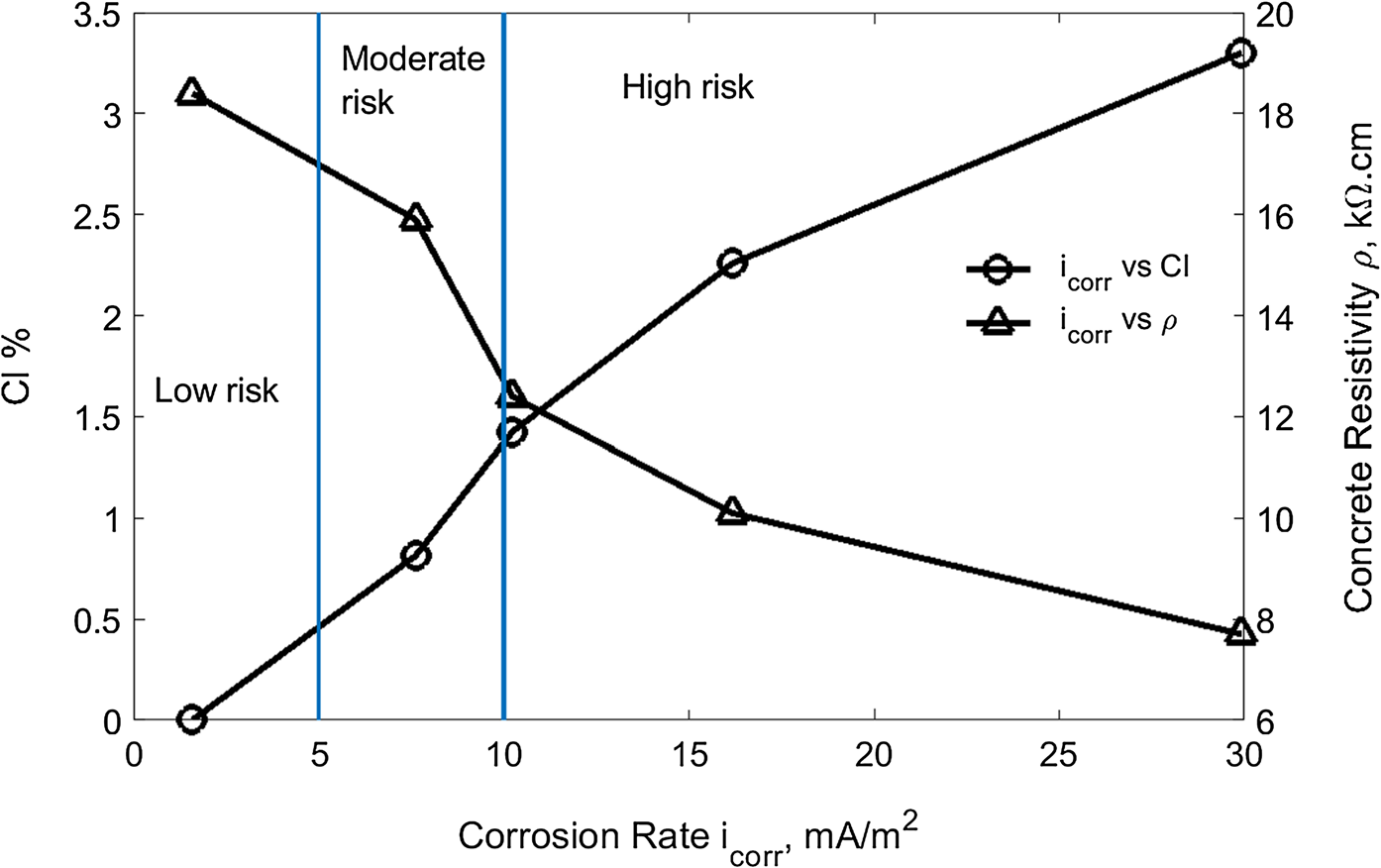
Reinforcement corrosion rate versus chloride contents and concrete resistivity

Figure 4 shows the correlation between the corrosion rate and chloride content and the corresponding concrete electrical resistivity for these specimens. It can be clearly seen that the higher chloride content or the lower the concrete resistivity, the higher the reinforcement corrosion rate. Previous research [34] suggested that corrosion risk is considered to be low when corrosion rate is in the range of 1–5 mA/m^2^, moderate when in the range of 5–10 mA/m^2^, and high when greater than 10 mA/m^2^. In terms of the classification, reinforcements will have a low corrosion rate if the total chloride content is less than 0.45% by the mass of cement and this chloride content may be taken as a threshold for the risk posed by reinforcement corrosion. If chloride content is over 1.4%, reinforcements present a high corrosion rate. The threshold value is in agreement with that recommended in literature [35], in which the critical chloride content was suggested in the range of 0.4–1% by weight of cement. In terms of concrete electrical resistivity, Fig. 4 shows that reinforcements will are likely to experience a low corrosion rate if the concrete electrical resistivity is higher than 17 kΩ cm, or a high corrosion rate if the concrete resistivity is less than 12.5 kΩ cm. The result is in agreement with previous research. For example, an earlier study [36] concluded that very high corrosion occurred when resistivity was less than 10 kΩ cm. Based on an investigation of the corrosion damage in a highway bridge, Cavalier and Vassie [37] also concluded that corrosion is almost certainly occurring when concrete resistivity is below 5 kΩ cm, but it is generally insignificant for values of resistivity above 12 kΩ cm. Another study considered that the risk of corrosion is negligible when resistivity exceeds 20 kΩ cm but becomes very high when resistivity is lower than 5 kΩ cm [38, 39].

### The effect of CP operation time on instant-off potential

**Fig. 5:**
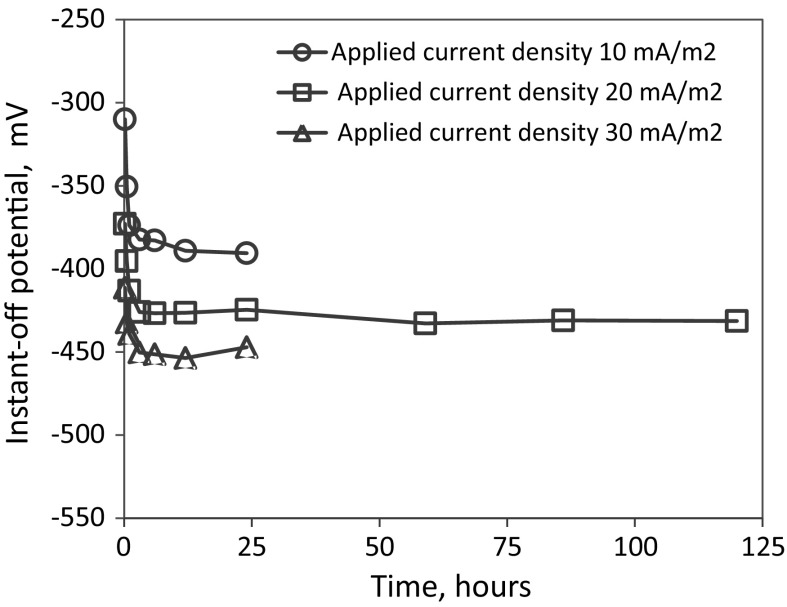
Reinforcement instant-off potential versus CP operation time

The potential of the reinforcements instantly after the interruption of CP is called the instant-off potential which, in the present study, was automatically measured in 1 s after the CP was switched off [18, 21]. Figure 5 shows the variation of the instant-off potential of the reinforcements as a function of the time of CP operation under different protection current densities for the specimens with 1% added NaCl. For the case of 20 mA/m^2^, the measurement were conducted for up to 120 h (5 days), while for the other two cases the measure were interrupted after 25 h. It can been seen that, for all the three cases, the instant-off potential displays a significant change in the first 3 h of CP operation under all the applied current densities. After 3 h, all the curves become flat, showing a very slight variation with time, suggesting that the system had reached a stable state. According to the results in the Fig. 5, all the parameters used for CP performance assessment, in this study, were taken after 24 h on CP implementation.

### Effects of CP current density and chloride content on instant-off potential

**Fig. 6:**
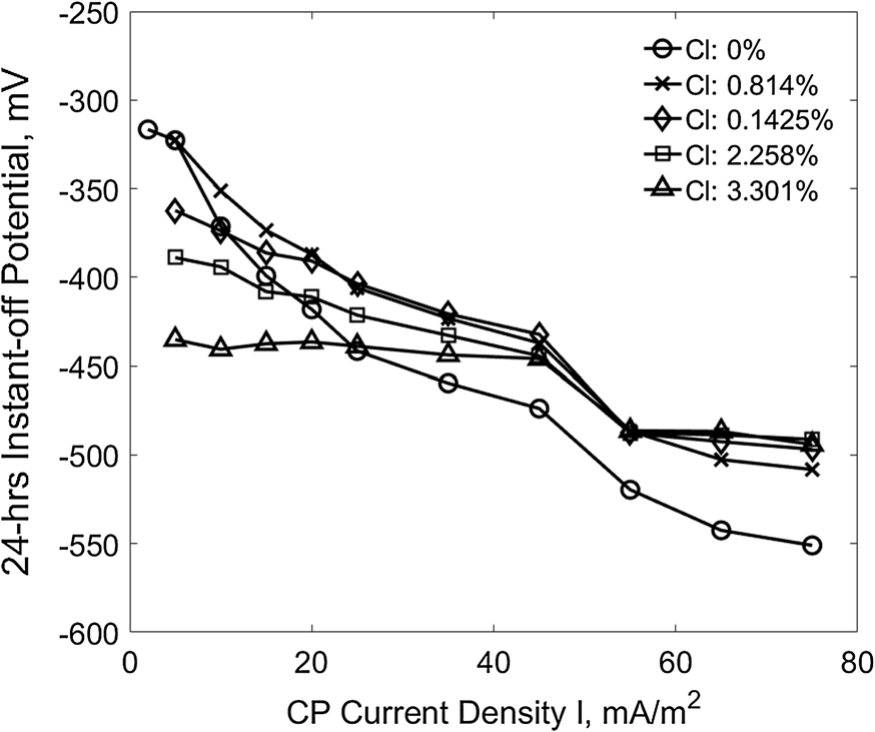
24-h instant-off potential versus CP current density at different chloride contents

Instant-off potential is one of the important criteria used to evaluate CP performance [18], for example the − 720 mV is recommend in British standard [21]. Figure 6 shows the variation of the 24 h CP instant-off potential of the reinforcements in the specimens of different chloride contents under different CP current densities. It can be seen that the absolute value of instant-off potential increases with the increase of the applied CP current density, the slope of the curve become flat when the concrete chloride content increases. From the result, it can be seen that even at the highest applied current density (i.e., 75 mA/m^2^) for all the different chloride contaminated specimens the − 720 mV criterion is still far away to be achieved.

### 4-h potential decay

**Fig. 7:**
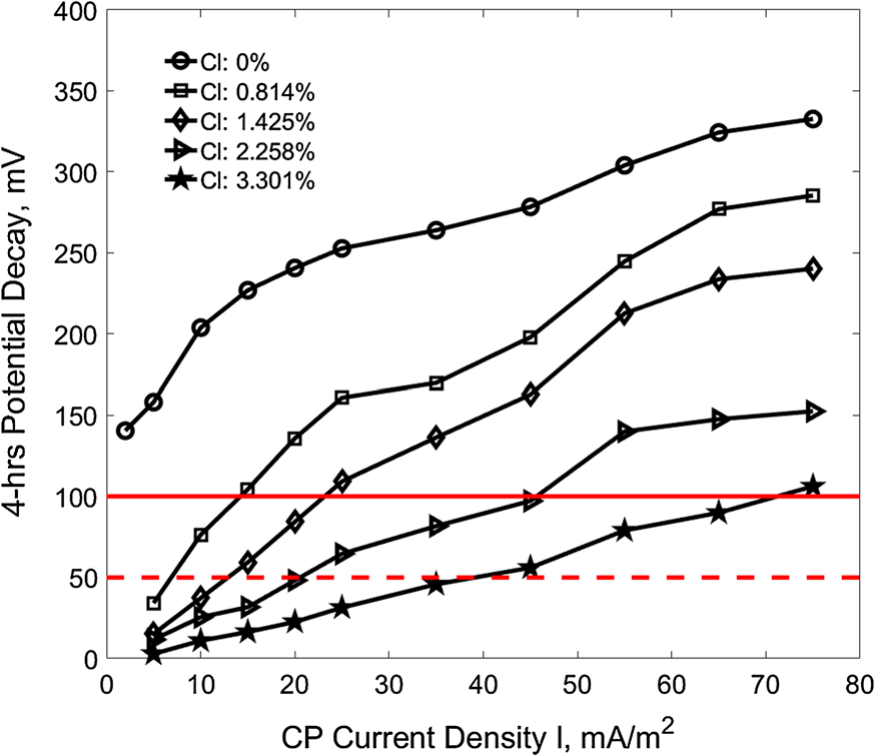
Reinforcement depolarization versus CP current density (the horizontal solid line for the criterion of 100 mV and the dash line for 50 mV)

The potential difference between the instant-off potential and the potential measured at the 4 h after switching off the CP current is called the 4-h potential decay which is another important parameter used to evaluate the effectiveness of CP operation [40, 41]. Generally, 100 mV depolarization in a 4 h period of time is the most accepted criterion. Figure 7 shows the variation of reinforcement depolarization (4-h potential decay) against the applied CP current density at different chloride contamination. It can be seen that for a certain chloride content, the depolarization of the reinforcements increases with the increase of the applied current density. For chloride free specimens, the 4-h potential decay curve is in the region above 100 mV (the horizontal solid line). It indicates that the reinforcements in a chloride free concrete environment are safe from corrosion even without CP (i.e., *I* = 0). Comparing the Figs. 6 and 7, it can be clearly noticed that, for example, a current density about 15 mA/m^2^ is sufficient to provide the required protection for the reinforcement in the concrete of 0.814% chloride in terms of the 100 mV potential decay criterion. However, current density of 75 mA/m^2^ is not enough to protect the reinforcement even in chloride free concrete in terms of the − 720 mV instant-off potential criterion.

**Fig. 8:**
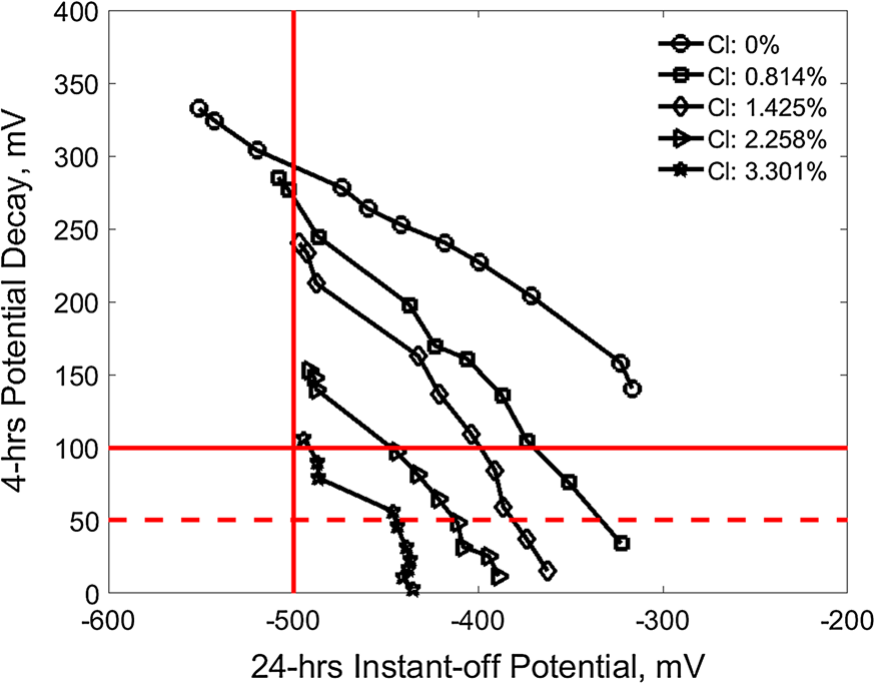
Reinforcement depolarization versus CP current density and 24-h CP instant-off potential (the horizontal solid line for 100 mV potential decay and the dash line for 50 mV while the vertical solid line for − 500 mV instant-off potential)

Figure 8 compares the two conventional criterion parameters, i.e., the 24 h CP instant-off potential and 4-h potential decay at different CP current density in terms of the results in Figs. 6 and 7. It shows that in terms of the 100 mV 4-h potential decay criterion, − 500 mV 24-h CP instant-off potential is sufficient to protect the reinforcements in all the investigated contaminated concretes.

**Fig. 9:**
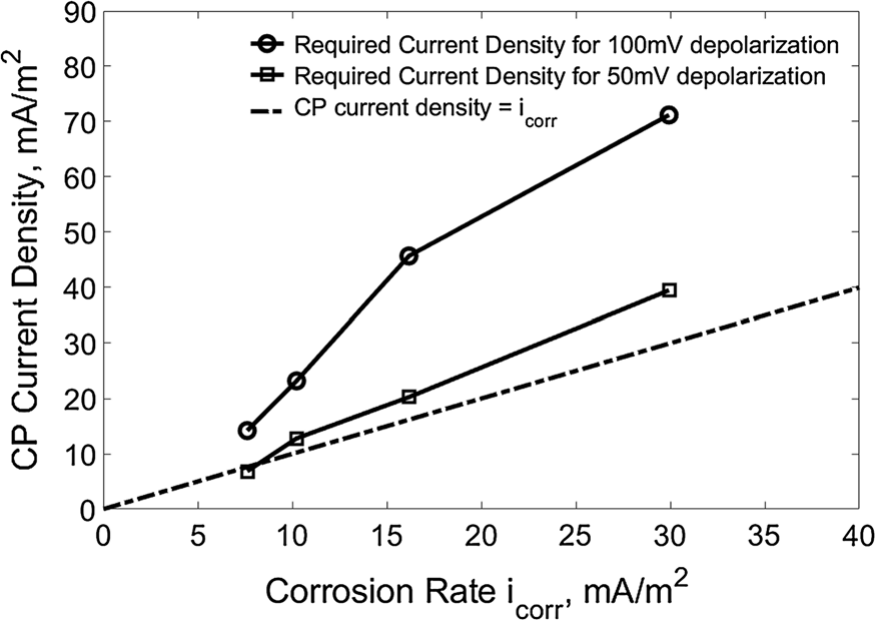
The required CP current density for different depolarization versus the initial corrosion rate of reinforcements

Figure 9 shows the required current densities for 100 mV (the interception points on the horizontal solid line in Fig. 7) and 50 mV (the interception points on the horizontal dash line in Fig. 7) depolarization (the 4-h potential decay) for the reinforcements at different initial corrosion rates before CP operation. The dash-dot line indicates the condition when the applied CP current density equals to the initial corrosion rate of the reinforcements. It can be seen that the suggested protection current density in terms of the 100 mV depolarization criterion is much higher than the corrosion rate of reinforcements. Particularly, the extra protection current density is projected at a high CP current density when reinforcement exposes to high chloride contamination or has a high initial corrosion rate. However, the CP current density in terms of the 50 mV depolarization condition is very close to the dash-dot line at all reinforcement initial corrosion rates.

**Fig. 10:**
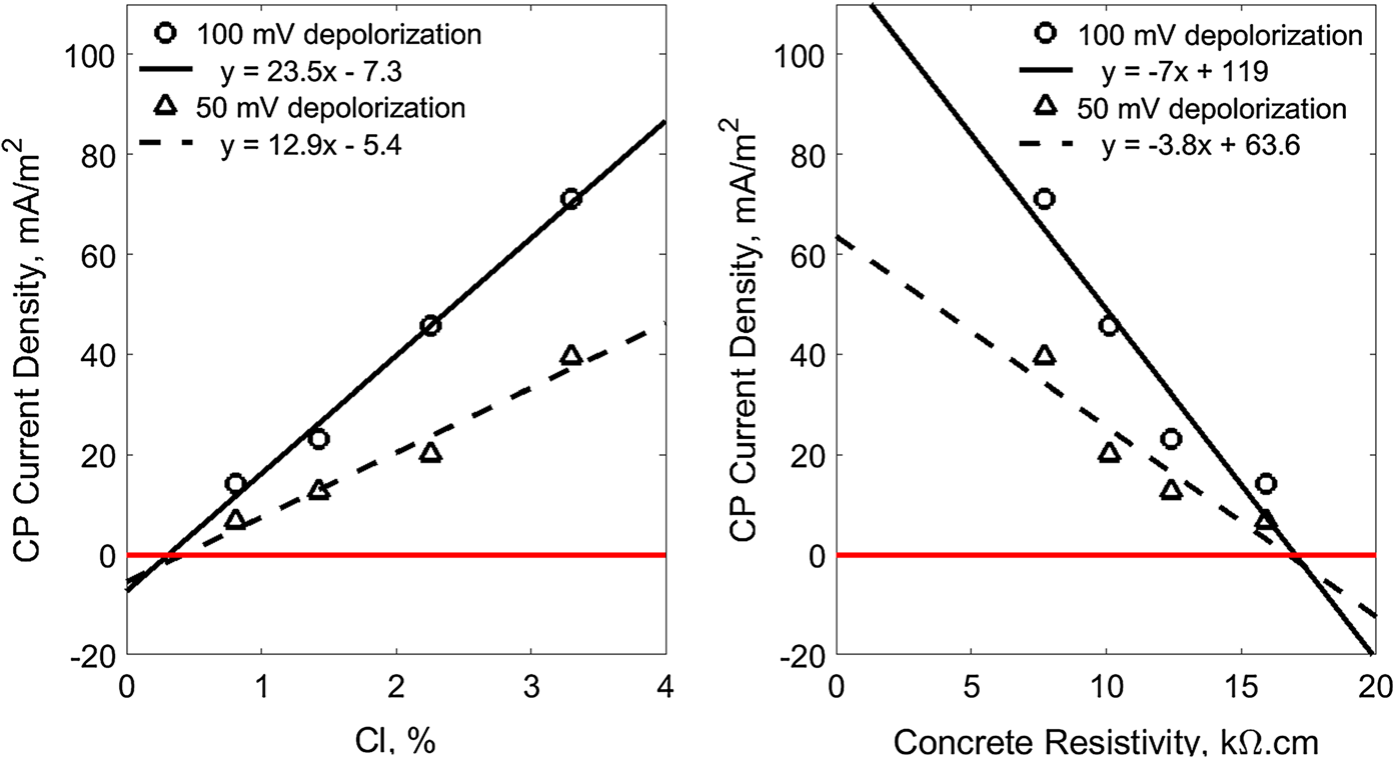
The required CP current density for the 100 mV and 50 mV depolarization versus chloride content and concrete resistivity (the horizontal lines indicate no CP current)

Figure 10 shows the CP current densities which give the 100 mV and 50 mV depolarization (the 4-h potential decays) for the reinforcements in the concrete of different chloride content and the corresponding electrical resistivity. The data are the interception points of all the curves in the Fig. 7 on the solid horizontal line at 100 mV and the dash horizontal line at 50 mV 4-hrs potential decay. It can be seen that the required CP current density for both of the 100 and 50 mV depolarization present an approximately linear correlation to the chloride content. A linear correlation to the concrete resistivity can also be assumed for practical purpose as well. According to the results, there is no CP needed when chloride content is less than about 0.31%, a value about 75% of the 0.45% the upper limit for low risk that was discussed in the Sect. 4.1 before according to the classification of Broomfield [34], or when concrete resistivity is more than about 17 kΩ cm.

**Fig. 11:**
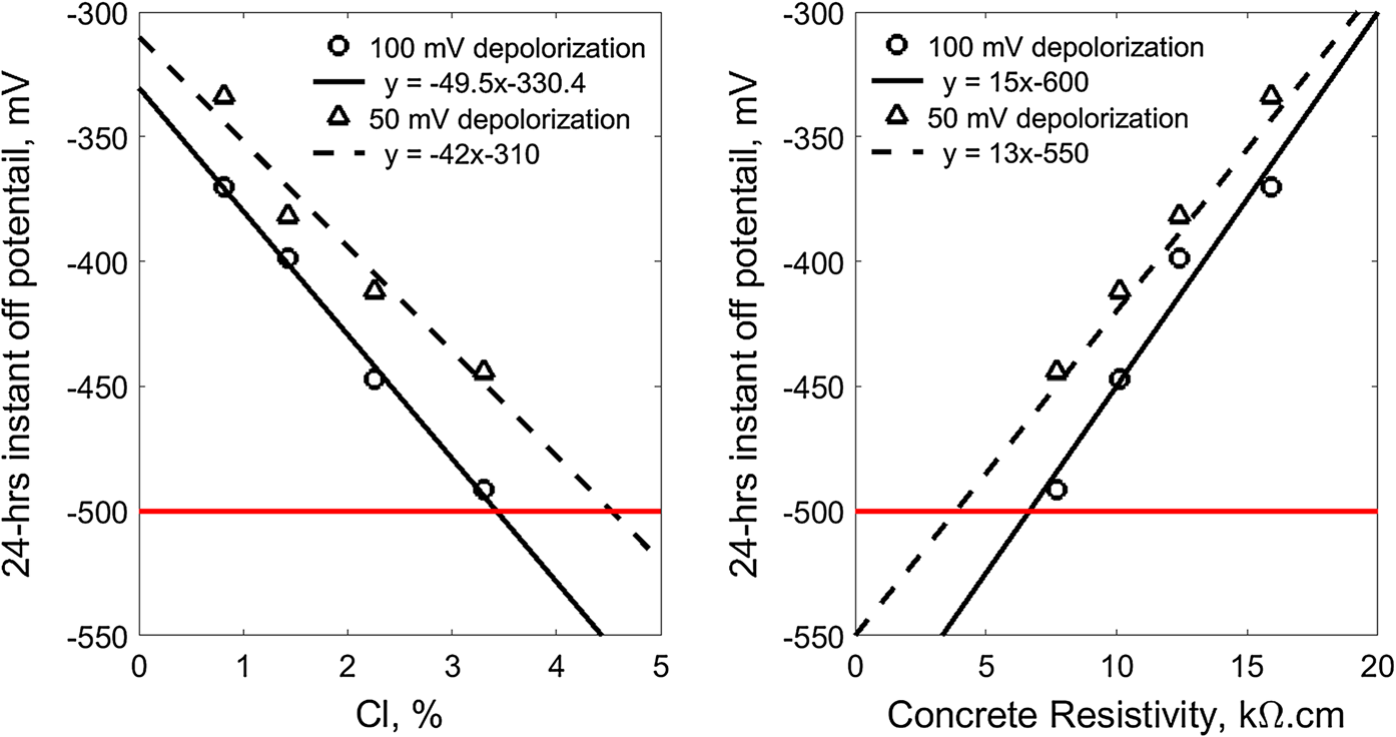
The required 24-h CP instant-off potential for the 100 mV and 50 mV depolarization (4-h decay potential) versus chloride content and concrete resistivity (the horizontal lines indicate − 500 mV instant-off potential)

Similarly, Fig. 11 shows the 24-h CP instant-off potential which corresponds to the 100 mV and 50 mV depolarization (the 4-h potential decay) for the reinforcements in the concrete of different chloride content and the corresponding electrical resistivity. The data are the interception points of all the curves in the Fig. 8 on the solid horizontal line at 100 mV and the dash horizontal line at 50 mV depolarization. It also demonstrates that the required instant-off potential for both of the 100 and 50 mV depolarization can also be approximately characterised using linear correlation to both the chloride content and concrete resistivity, respectively. It can be seen that − 500 mV instant-off potential can provide adequate protection for the reinforcement in concrete of up to 3.4% chloride content or of more than 6.7 kΩ cm resistivity in terms of the 100 mV potential decay criterion. However, taking 50 mV potential decay as a criterion, the − 500 mV instant-off potential can provide sufficient protection for the chloride content up to 4.5% or concrete resistivity less than 3.8 kΩ cm.

Finally, for the results obtained in this study, there are two specific concerns that should be clarified:

1. The initial formation of passive layer was not taken into account, as chloride was added into the mix water to accelerate corrosion. Without considering the initial passivation, the measurement obtained in this work can only provide the guidance for the reinforcements that have already been experiencing active corrosion, such as, for example, those in chloride contaminated concrete with a low pH pore solution.
2. All measurements are based on the hypothesis that corrosion on the rebars is homogeneously distributed, i.e. even if the corrosion might be localized on the microscopic scale, on a macroscopic scale there are no regions where corrosion is substantially more severe compared to others. In order to work under this assumption, in preparing specimens, efforts were made to enhance even chloride distribution in concrete by (1) adding Cl into mixing water as the most conventional measure [42–46] but also (2) curing samples in water containing the same amount of chlorides. The examination of the rebars after CP measurement had confirmed the corrosion took place on the whole exposed rebar surfaces.

## Conclusions

This paper has produced a number of experimental data for CP performance on the reinforcement of Portland cement concrete exposed to chloride contamination under atmospheric condition. From the reported work, the following conclusions can be obtained:

1. A total chloride content of 0.31% by weight of cement or 17 kΩ cm concrete electrical resisitivity may be set as a threshold for CP implementation to protect the reinforcements in Portland concrete from corrosion.
2. An instant-off potential of − 500 mV with respect to Ag/AgCl/0.5KCl electrode can provide adequate protection, in relation to the 100 mV depolarization criterion, for the reinforced concrete of up to 3.4% chloride contamination by weight of cement, or concrete resistivity is no less than 6.7 kΩ cm.
3. A clear correlation between CP current requirement and chloride content and concrete resistivity were obtained, and characterisation modelling has been suggested.
